# What Are the Best Materials for Filling Children's Teeth

**Published:** 1899-08

**Authors:** C Edmund Kells

**Affiliations:** New Orleans, La.


ARTICLE III.

### WHAT ARE THE BEST MATERIALS FOR FILLING CHILDREN'S TEETH.

C EDMUND KELLS, JR., D. D. S., NEW ORLEANS, LA.

A five-minute paper upon such a prolific object must of necessity be concise, hence to the practical treatment of the temporary teeth at once.

These will be divided, from a clinical standpoint, into classes : A, good: B, bad; C, very bad.

Class A. Good teeth, of course, are those to which, profitable by our teachings, the mother has given good care and the little patient brought to us at an early age, say two and a half years. At this period the teeth are in perfect order, clean, and pleasing in appearance.

The patient is examined every three or four months, and from time to time a spot of decay may appear in the crowns of the molars, which is filled at once with amalgam, however tiny the cavity may be. At the same time, whenever necessary, all the exposed surfaces are polished with orange-wood and pumice, or the engine and a rubber cup may be used. The approximal surfaces are also kept bright by pumice and floss silk.

The incisors, under this care, remain perfect until shed.

The treatment these teeth receive is mostly preventive, and the time (and money) so spent is well invested.

Class B. These patients are brought to us probably after a night of suffering, and say at five years of age, and bespeak at once the neglect they have endured with compound approximal cavities in the molars, pulps nearly or quite exposed, and perhaps an abscess in progress of formation. The incisors also may be involved. The patient is not a type of good health, but nervous, and afraid of everything.in the office, ourselves included.

The first step to be taken is to gain the child's confidence, which can only be accomplished by inflicting no pain at the first visit. Some small crown cavity (usually to be found) is slightly prepared and filled with cement, and the patient is greatly encouraged by the idea of having had a tooth filled without pain. By the time all of the other work is completed this filling (?) is to be removed, the cavity properly prepared and filled with amalgam.

The large compound approximal cavities are in due time prepared as thoroughly as possible, care being taken not to exposs the pulps; they are cauterized one or more times with silver nitrate, filled with amalgam if safe, or, if not, with a chlorid of zinc cement. If all progresses smoothly, at the end of six months or a year they may be refilled with amalgam.

Sometimes, when the teeth are crowded, after cauterization, gutta-percha is packed into two facing compound approximal cavities, and allowed to remain for a month or more, when a filling can be more satisfactorily inserted.

If the pulps are exposed after the use of the silver nitrate a cream of oxid of zinc and creasote is flowed over the exposed portion previous to the insertion of the filling.

These pulps, if thoroughly exposed, will die, but probably painlessly, and the tooth may remain comfortable for some time.

Any tooth in this condition at the age of five will not survive its period of usefulness, but must be lost sooner. However, if the second molars can be held in place, at almost any cost, until the first permanent molars have come through and are well articulated, this should be done, after which their loss is not so serious.

It is not my practice to fill the root canals of temporary teeth, consequently the treatment of these teeth is merely palliative; when that fails to give relief, extraction is resorted to.

Class C. Under this we will describe a poor little suffer of two and a half years of age, with all the teeth

"rotted to the gums," as we are told, and which description is in fact more accurate than elegant. True enough, the crowns are gone, but in many the pulps are still alive and exquisitely sensitive. Here nothing can equal the cauterization by silver nitrate, which, repeated at intervals, will allay the sensitiveness, and is all that can be done for the little sufferer.

When a girl who has been under my care since childhood marries, she is impressed with the necessity of caring for her children's teeth. She is taught to brush, not rub with a rag, the infant's teeth from the time of their appearance; at first with clear water, and later, when the child is old enough not to attempt to swallow everything that is put in its mouth, with prepared chalk. Floss silk should also be passed between them daily, and when the child is old enough the mouth should be thoroughly rinsed with diluted lime water night and morning. This constitutes the care the mother is instructed to give the temporary teeth, besides which they should be sent to me for an examination three times annually.

*The Permanent Set.*—The judicious treatment of the first permanent molars is of paramount importance. In my practice there is a tendency to crowded arches, and very frequently the extraction of four teeth becomes advisable.

If the first permanent molars erupt in a defective condition, which they very frequently do, and break down immediately, they are nursed along until the patient reaches the age of eleven, and then extracted. Removing them at this age leaves the twenty anterior teeth for mastication, and allows of the eruption of the second molars in in a perpendicular position and almost in the places of the first molars. Meanwhile the bicuspids have dropped back a lttle, and the entire arch at the age of fifteen is roomy and in good condition.

If the first molars are removed after the appearance of the second molars these are sure to tip forward more or less, and the crowded condition of the anterior teeth is not so readily overcome, and, altogether, a bad result is sure to obtain.

For nearly twenty years the judicious extraction of the first permanent molars has been practiced by me, and the satisfactory result so obtained is a daily object-lesson among those patients who are still in my care.

When the first molars are good, one set of the bicuspids, either the first or second, are oftentimes removed with the best results.

As the teeth of the permanent set are erupted they are watched carefully, at least two visits a year being insisted upon. The smallest pits that penetrate through the enamel are filled at once. If these occur before the teeth are fully erupted they are filled with oxychlorid of zinc first, and later with amalgam or gold. If the teeth are soft, gold is never used. Amalgam is a very satisfactory material for the fissures of bicuspids of children.

Sometimes these buccal and fissure cavities are quite deep, though the enamel-margins are quite hard; in which cases the cavities are lined with either gutta-percha or cement, usually the latter, and gold or amalgam completes the filling.

When all is ready for the filling the cavity is swabbed out with creasote (Morson's only), and then dried with hot air. This will leave the walls in a glistering condition; and if any spot of decay or softened tissue remains will at once discolor it, and it is removed.

When very deep cavities are found the enamel borders are prepared thoroughly, but the decay over the pulp is not entirely removed for fear of exposing the same. This is coated with a cream of oxid of zinc mixed with creosote, and dried and covered with oxychlorid. Later a part of the cement is cut out, and amalgam or gold packed over the remaining protecting layer.

The oxychlorid of zinc is a treatment of the tooth under which the tooth will improve. The zinc phosphate is a filling-material only, porous to a certain extent, and does dot benefit the tooth otherwise thus serving as a filling material.

The approximal surfaces are watched carefully, and upon the slightest suspicion the teeth are wedged apart and the discoloration removed with fine polishing strips; after which the surfaces are brought to a high polish by the use of lamp-wick carrying moistened pumice. A carrier for this purpose, devised by my father some thirty or more years ago, I have never seen equaled, so I present one for your inspection.

Approximal cavities in children under fifteen years of age are never filled with gold, but with oxychlorid of zinc. Usually the dam is applied, the fillings inserted, patient seated in another room with a book or magazine for from forty-five to sixty minutes, the meantime being employed on other patients, and so is not wasted. The cement is then carefully finished, and paraffin burnished on with a hot instrument.

It is not unusual to find at the age of twelve or thirteen years cavities on the approximal surfaces of all the lower incisors, and even cuspids. Experience and observation have taught me long since that gold will not save such teeth at all, while the above treatment will. I have during this past month filled such teeth with gold for a miss of nineteen years, for whom they were filled with cement in May, 1888, or ten years ago, the cement fillings having wasted but slightly during all this time, and the teeth in the best of condition.

It is essential that a child be taught how to brush the teeth properly, and for this purpose I keep in my cabinet a tooth-brush and set of teeth mounted on continuous gum, and with which I give a careful object lesson.

Finally, when all other work is completed, the teeth are thoroughly cleansed and polished, and great stress laid upon the necessity for the patient's keeping them in that condition.

I am told that the teeth of children in this section will not stand the same treatment as those in the North. Of this I know nothing personally, but of the utter failure of gold in children's teeth I do not judge by my own work alone. Scores of my litile patients go off to school, and have gold fillings inserted by operators of the best reputation, which, however, soon fail. So my hearers of a distant clime must please bear climatic considerations in mind when criticizing my methods, and also my work when it falls under their view.

H. S. AMBLER, D. D. D., M. D., CLEVELAND, O.

Tin has physical properties which render it desirable as a filling material; it is about one-fourth as tenacious and malleable as gold. Again, gold is nearly four times as good a conductor of heat, and six times as good a conconductor of electricity; and its capacity for absorbing heat is nearly twice as great. On account of its pliability it is easily adapted to the walls and margins, and a perfect fit is made, thus preventing capillary action and preventing further caries. Of all the metals used for filling, it is the best tooth-preserver and the most compatible with tooth-substance, and it does not change form after being packed into a cavity.

Tin possesses antiseptic properties which do not pertain to gold for arresting caries in imperfect teeth, and, owing to its therapeutic quality, and being a rather poor conductor, there is a strong probability of calcification taking place under it.

In a few cases tin does not discolor, in others it presents a grayish appearance, but in the majority it is more or less blackened on the surface, the metallic oxid penetrating the dentin slightly and acting as a protection, because it is insoluble; but in either event the tooth is preserved. If the cavities were sensitive when filled with tin, and in future years it is removed for the purpose of filling with gold, it is generally found that the sensitiveness has disappeared.

Many failures are made in filling the anterior teeth of children with cohesive gold, but if we work as carefully and try as faithfully with tin, good results would be produced and many teeth would be saved; and; as tin can be cut easily, more cervical margins would be finished smoothly. The objection that tin is too soft does not have any great weight when used for approximal fillings which do not include the incisive edge or occlusal surface.

For some time we have been using foil, prepared according to our suggestions, manufactured by The S. S. White Company as a special product; with it a filling can be made which is nearly twice as hard as the ordinary tin filling. This foil is more tenacious and cohesive than any we have ever seen. For eight months it has given complete satisfaction in the Western Reserve University Dental College clinic, and in the technic work it has entirely superceded gold foil.

The idea that gold is the best filling material in all cases has been completely refuted by the new "New Department creed;" therefore it is our duty to fairly consider and faithfully try other materials, to the end that we as dentists may be able to save a larger number of carious teeth.

Good tin foil, in proper condition; is cohesive when force is applied, and can be used for filling in the same manner in which cohesive gold is used; and a tin filling properly condensed, layer by layer, is a solid mass which can be cut or filed. Cavities are generally prepared the same as for gold, then a stripe of tape, narrower than the orifice, is folded once or twice at the end and placed where the filling is to be commenced, the tape being folded back and forth as the operation progresses by hand-mallet or hand-pressure; the finishing to be completed the same as for gold. For filling with a hand mallet use instruments with medium serrations, and a steady, medium blow with a four-ounce mallet; in force of blow we are guided by thickness of tin, size of plugger, depth of serration, strength of cavity walls and margins. The best results are only obtained by having absolute dryness.

Filling the anterior teeth of children with tin has been practiced by the writer more or less for thirty years, and he is convinced that such operations are practical and durable, and will last from five to twenty years, according to the quality of tooth-structure, size and location of cavity, care of the teeth, environment, and care with which the filling is inserted. In "Tin Foil and its Combination for Filling Teeth," recently published by the S. S. White Company, cases are recorded where tin fillings have lasted forty years.

Up to the age of fourteen years we find many teeth which are imperfect in structure; in such cases we advise tin, for as the patient advances in years the tooth usually becomes better, so that, if desirable, the fillings can be removed and saving operations made with gold. By treating cases in this manner very little, if any, tooth-structure is lost.

In filling these anterior teeth we suppose the cavities have been taken before they involve the incisive edge, but if the edge is involved the tin should only be rounded out, no attempt being made to restore a corner.—*Cosmos.*

---

ARTICLE IV.

